# Risk factors for PTSD of Shidu parents who lost the only child in a rapid aging process: a cross-sectional study

**DOI:** 10.1186/s12888-020-2446-3

**Published:** 2020-01-30

**Authors:** Qianlan Yin, Huaihui Zhang, Zhilei Shang, Lili Wu, Zhuoer Sun, Fan Zhang, Yaoguang Zhou, Xiangrui Song, Weizhi Liu

**Affiliations:** 1 Faculty of Psychology and Mental Health, Navy Medical University, 800 Xiangyin Road, Shanghai, 200433 China; 2 Shanghai Yangpu Mental Health Center, Shanghai University of Medicine & Health Sciences Teaching Hospital, Shanghai, 200093 China; 3 Lab for Post-traumatic Stress Disorder , Faculty of Psychology and Mental Health, Navy Medical University, Shanghai, 200433 China

**Keywords:** Shidu, PTSD, Aging, Older adults, Bereavement, Risk factors

## Abstract

**Background:**

The elderly population is rising globally, especially in China where a large population base causes the largest number of older adults in the world. Notably, Shidu people who are over the age of 60 and have lost their only child have drawn great public attentions as they become more elderly, medically unstable and worse mentally unhealthy. Posttraumatic stress disorder (PTSD) is one of the most common consequences resulted from the loss of the only child. However, few previous studies have examined PTSD in Shidu older aldults, and the risk factors are a relatively understudied area. Our study aims to estimate the prevalence and potential risk factors of PTSD and improve the possibility of early identifying the high-risk Shidu parents with PTSD, and successively provide timely and effective interventions.

**Method:**

Based on the register of population statistic information provided by the health family planning commission, 149 participants were enrolled randomly. Data was collected by interviews and questionnaires. Socio-demographic and bereavement-related information and physical health outcomes were obtained. PTSD Checklist-Civilian Version was used to screen for bereavement-related PTSD.

**Result:**

The morbidity of PTSD reached 30.9%, while in the group of age over 60 the morbidity reached 31.6%. Stratified by potential demographic risk factors, SDPs have significant between-group differences of PTSD. Specially, being women, higher income, losing the single child at older age, more serious medical conditions and being Shidu for a shorter period indicated higher severity of PTSD in SDPs. The single child dying at a older age and from accidence were also significant indicators. Regression analysis showed the gender of SDPs, hospital visits, and the cause of child death significantly predicted the severity of PTSD.

**Conclusion:**

With the accelerate process of aging, especially in China, Shidu older adults become a group deserved more attentions. PTSD is clearly a possible reaction to the loss of the only child. The gender and hospital visits of the Shidu older adults and the causes of their child’s death significantly related to the prevalence of PTSD, which could help to improve the possibility of early intervening.

## Background

Population aging is one of the most important demographic phenomena driven by fertility decline and the continuing extension of the life expectancy. According to the new standards that countries enter the aging society if old people exceeds 7% the national population, now 91 countries in the world have met the standards and the highest degree of aging is in Japan with 26.02% people over 65 years old, followed by Italy with a percentage of 22.36% (numbers cited from United Nations population and social programme population data in 2015). In terms of the future trendy, aging population of the United States is projected to double by 2030 to 72 million adults, representing 20% of the total U.S [1]. The population of older adults is rising globally, however, similar trends are also in the rapidly developing countries. In China, although the trend started in the twentieth century later than developed western countries, but the process of aging is fast and steadily driven by the rapid economical development and, additionally, China also has the largest number of older adults as to a large population base. According to the researchers’ expectation, by 2030 Chinese old people will account for 24.6% of the world’s population aged 60 and above [2]. Notably, a special cohort old Chinese people, referred to “Shidu” old adults, who are over the age of 60 and have lost their only child (Shidu is the Chinese transliteration for ‘losing the only child’ and over 60 is an acknowledged age distinguishing the young and the old due to the retiring age in China set at 55), have aroused great concerns in the society. The majority of these people with the one-child family in response to Chinese family planning policy in the early twentieth century have been into their old age without their only child, who is supposed to act in the role of the care provider to their aging parents. Today, the SDPs (Shidu parents are parents who have lost their only child, abbreviated to SDPs) are becoming more elderly, medically unstable, and more likely to require care from family and social institutions. Researchers have estimated the population size of family with only child aged 15–29 years in China and roughly predicted that every year in our country at least 82,460 people aged between 15 and 29 years old died, putting in anther way, 82,500 families lost their only child and 165,000 Shidu older adults would emerge annually [3]. Therefore, the number of families with the loss of the only chcild in China will increase gradually along with the process of aging and the aggravation of the trend of low fertility rate, which underlies the social endowment issues.

However, the financial and social dilemmas are not only the substantial problems of the Shidu old adults but also the great grief of the loss of the loved child. Combined with having no social support from children, experiencing inequality within the social security system, witnessing a traumatic event and psychological suffering from losing a child, and being marginalised and sometimes ignored by the society, SDPs have higher vulnerability to physical and mental health problems [4]. An investigation showed that only 11.4% of interviewed SDPs maintained the comparatively normal psychological status [5]. Depression and anxiety are of high incidence in SDPs and commonly showed soon after the loss. Most of them remain in the grief of loss and spiral into symptoms of Prolonged Grief Disorder (PGD) and/or Post Traumatic Stress Disorder (PTSD) [6, 7]. According to recent studies, PTSD is prevalent and serious in Shidu parents. PTSD self-rating scale was used to investigate 105 patients who had lost their only child and found that 73 patients (69.5%) suffered from PTSD [8], and 173 SDPs were investigated at a time when the child died at least 5 years, finding that there were 27.7% mothers and 12.5% fathers with PTSD [9]. Even after 18 years-a longer period of the child’s death, Christiansen et al. found there still existed PTSD symptoms in the SDPs [10]. Consistently, we found SDPs had a higher risk of the development of PTSD compared to the parents with a living child, and the rate of PTSD in the Shidu group (*n* = 95) was up to 32.6% in a published research of our team [11]. Regarding the previous studies, the prevalence of PTSD in older people ranged from 4 to 14% following traumatic experiences such as natural disasters, physical injuries, and threat of illness [12–14]. However, trauma and PTSD have been hidden in the lives of older adults as the related clinical information is not typically recognized, acknowledged or shared by the older patients [15]. There is comparatively less research on trauma and PTSD in the older adults compared with the middle-aged and younger, and less report on the trauma of losing the only child which is referred to one of the most stressful events for the older adults [16]. Clinical lore indicates that the older adults may not recognize or admit to difficulties due to the concerns of the stigma of being Shidu in Chinese culture and health care professionals may under recognize or under treat these conditions. Hence, the highlight of the research on PTSD in Shidu older adults, especially on the risk factors related to the genesis of PTSD, are conducive to better understand the special consideration and intervention for this population.

The risk factors of PTSD in Shidu older adults are a relatively understudied area. Referred to other bereaved studies on the elderly people of different bereavement like spouse and relatives, we found some significant predictors were reported such as the individual difference including gender, peritraumatic, and psychosocial factor, and the characteristic of the dead person [17]. Cohen-Mansfield J et al. reported a gender-differential effect of the parental bereavement on 20-year follow-up mortality of older people [18]. Feng Z reported being female, illiterate/semi-literate, never married, divorced or widowed, and without a pension after retirement were positively associated with poor mental health in the older adults without child [4]. Subjective physical health is thought to reflect psychological adaption. Therefore, the healthy condition of bereaved people should be considered [9, 19]. However, there was no reported evidence for the association between the characteristics of dead child (like the age and the gender of them) and PTSD, yet death causes of the only child might also have relation to the morbidity of PTSD testified by Chan et al. and Xu et al. [20, 21]. They reviewed bereaved parents who had lost children in sudden and unexpected accidents were at a high risk of suffering PTSD as one of the most common psychological and social consequences. Therefore, to further explore the potential risk factors of PTSD in the Shidu older adults, we investigated a group of the aging SDPs, who were unique and unaccessible, to estimate the prevalence and potential risk factors of PTSD. We hypothesized that the gender, economical state and medical state of the SDPs and characteristics of the death of the only child could be significant risk factors for PTSD. The aim of our study is to improve the possibility of early identification of the high-risk Shidu older adults with PTSD, and providing timely and effective interventions.

## Method

### Participants and procedure

The information about the SDPs from the Register of Population Statistics provided by the Health Family Planning Commission has been collected. The inclusion criteria were as follow: 1) both SDPs were above 49 (which is thought to beyond the female reproductive age) or they do not want another baby; 2) the participants had only one biological child and the child had died at least 1 year ago; 3) the participants have not adopted a child by the time they participated in this study; 4) individuals with serious mental disorders such as schizophrenia diagnosed by the psychiatrist were excluded. 155 out of 800 Shidu families who met the inclusion criteria were randomly selected from 11 communities of a district in Shanghai by stratified random sampling (only one parent from each Shifu family was selected as our participant). With the help from local social workers who knew the families and provided a brief description of the study, 149 participants were agreed to sign the written informed consent and completed the face to face interviews, approximately lasting 40 min. Data were collected between September 2015 and December 2016. Descriptive data of the total sample are presented in Table 1.

**Table 1:** Demographic information and bereavement-related information of PTSD in SDPs. Note: PCL-C=Post-trauma stress disorder checklist-civilian version

	N	PCL-C	Z/Chi-square	p
Gender			-4.041	0.000***
Male	59	35.85 ± 14.72		
Female	90	47.24 ± 16.60		
Age			−0.401	0.682
< 60	35	43.00 ± 16.17		
> =60	114	42.65 ± 17.04		
Income			−2.659	0.008**
Below 3000	54	40.12 ± 17.05		
Above 3000	95	47.33 ± 15.41		
Education			−0.560	0.575
Junior or below	74	42.03 ± 17.34		
Senior or above	75	43.43 ± 16.31		
Marriage			1.162	0.762
Not divorced	124	42.46 ± 16.80		
Divorced	4	43.26 ± 20.69		
Married again	7	49.63 ± 19.67		
Widowed	14	43.43 ± 15.20		
Religion			1.184	0.553
none	122	42.25 ± 16.63		
Buddhism	21	46.48 ± 17.22		
other	6	39.50 ± 19.77		
Age of being Shidu			4.190	0.123
Less than 50	60	38.87 ± 15.21		
50–60	78	45.22 ± 17.28		
Above 60	11	46.18 ± 18.87		
Years of being Shidu			7.782	0.051
< 5	33	48.33 ± 16.46		
5–10	42	43.17 ± 18.89		
10–15	34	43.62 ± 16.19		
> 15	40	36.90 ± 13.71		
Annual hospital visit			17.67	0.000***
≤ 2	38	34.45 ± 16.31		
3–5	23	43.74 ± 19.69		
6–9	16	40.19 ± 14.14		
> =10	72	47.35 ± 15.05		
Medical conditions (number of physical problems)			12.434	0.002**
< 1	47	37.00 ± 16.92		
1–3	77	43.19 ± 15.76		
> 3	25	52.08 ± 15.72		

*Note.* ** means *p* < 0.01, and *** *p* < 0.001

This project was approved by the Ethics Committee of Navy Medical University. A team, comprising a psychiatrist, a psychologist, and several psychology graduate students, conducted the face-to-face interview with SDPs and provided necessary support when asking about sensitive items. The psychiatrist and psychologist were responsible for rating the questionnaire of PTSD and the psychology graduate students collected the household survey. Some participants became emotional during the interview, and then the experiment was paused to comfort them, or allow them to finish the investigation at another time.

### Measure

Socio-demographic information includes: gender, age, marital status, educational background, religious belief, family income and subjective assessment of family economic status comparing with the the average monthly household income in Shanghai in 2015. The loss-related variables included gender and age of the dead child, cause of death, time since loss, and age of parent when losing child.

The questionnaires for physical health outcomes were the same to the previous study [11]. Two items were all included. One items designed in form of multiple choice for collecting the number of diseases that SDPs have suffered. For further analysis, in this study, the health state of the SDPs was ranked in 3 levels according to the total number of their diseases (0 = 0 disease, 1 = 1 to 3 diseases, and 2 = 3 or above). The other item was a blank-filled question about the annual hospital visit times ranked in 4 levels.

PTSD Checklist-Civilian Version (PCL-C) was adopted to single out bereavement-related PTSD, which were developed by Weathers et al. [22]. The Chinese version of the PCL-C has been validated and widely used in studies [11, 23]. It requires respondents to indicate the degree to which they have been bothered during the past month by the PTSD symptoms according to the DSM-4th Edition. 17 items are on a 5-point Likert-Type scale include three factors: intrusion, avoidance and numbing, and hyperarousal. In our cohort, a cut-off score of 50 has been used to suggest a PTSD diagnosis with proper sensitivity and specificity, which was in the same reference range in clinical sampled studies [24, 25].

### Data analysis

155 in person interviews were conducted and collected 149 valid questionnaires. These 6 participants withdrew from the study because they could not control their grief and emotion during the the interview with the help of our psychiatrists and declined to continue the recalling and evaluation. Besides, 2 of them said they had been experienced recent traumatic events in last year according to their answer of the question about traumatic experience in the basic information questionairre. Therefore, to maintain the validity of our data, these 6 subjects were excluded and the residual subjects finished all the questions completely. Analyses were conducted using IBM Statistical Package for Social Sciences version 24. The distribution of PCL-C scores was slightly skewed to the right tested by Kolmogorov-Smirnov test and Shapiro-Wilk. Hence, non-parametric tests were more appropriate. The sample data then were divided into two groups by additional grouping variables. Z value and Chi-square were calculated for the further analysis of risk factors with the discrete PCL-C scores. The bivariate analysis was conducted to reveal the potential positive or negative relationship between significant categorical variables and the rate of PTSD. Finally, the logistical regression analysis was performed to explore valid predictors to PTSD. The group difference analysis and the bivariate analysis went with the report of standard errors. As in the regression, the test of bootstrap and calculation of the estimation interval was properly performed [26].

## Result

### Demographic information and bereavement-related information of PTSD in SDPs

76.5% of the cohort aged over 60, with the average age of 62.25 (Std. Deviation = 4.88), 50 as the minimum and 75 as the maximum. The average score of PCL-C was 42.73 (Std. Deviation = 16.79). Table 1 presents the rates of PTSD according to the demographic information. Different gender(Z = − 4.041,*p* < 0.001) and incoming groups (Z = − 2.659, *p* = 0.008) had significant disparity in PCL-C scores, while the other demographic factors such as age, education, marriage and religion had no significant difference in the rates. Moreover, participants with different ages of being Shidu showed no statistically significant differences in the rate of PTSD, and their years of being Shidu had nearly significant effects in the results of PCL-C (Z = 7.782, *p* = 0.051). Stratified by annual hospital visits and medical conditions, the differences of the rate of PCL-C were significant between groups with the *p* value of 0.001 and 0.002 respectively.

**Fig. 1:**
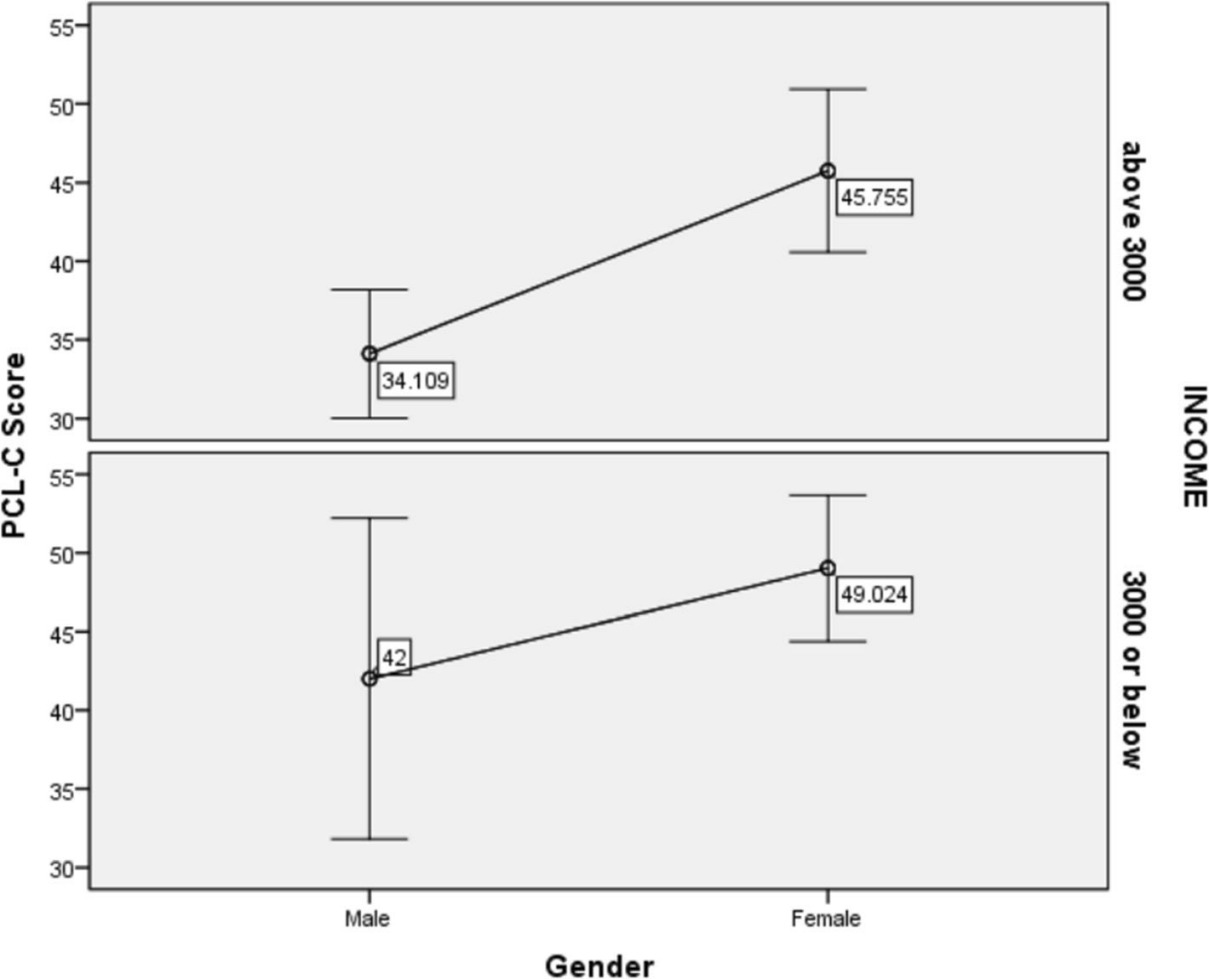
the comparison between gender and income of SDPs on the PCL-C score. Note: PCL-C=Post-trauma stress disorder checklist-civilian version. SDPs = Shidu parents

In the further analysis of differences, the SDPs in to PTSD group (*n* = 46, Mean_PCL-C_ = 63.67 ± 8.02) and non-PTSD (*n* = 103, Mean_PCL-C_ = 33.38 ± 9.72) group were dichotomous according to the clinical cut-off of 50 in PCL-C scale. In this case, the morbidity of PTSD in our cohort reached 30.9%. Figure 1 shows the compared results of the factors including gender and incomes as binary variables between the two groups. Sequentially, female SDPs (37 in 90) were significantly more likely to develop PTSD than males (9 in 59) (OR:3.816.7978, 95% CI:1.70–8.847, *p* = 0.01), the group with less than 3000 yuan income (23 in 54) were significantly more likely to develop PTSD than that with above 3000 yuan income (23 in 95) (OR:2.323, 95% CI:1.136–4.748, *p* = 0.027). Females of low income (the prevalence of PTSD was 46.2%) had significantly higher risk to PTSD than males of income above 3000(the prevalence of PTSD was 8.7%), and the value of OR reached 9.00 (95% CI = 2.702–29.983, *p* < 0.0001). Meanwhile, annual hospital visits (chi-square = − 2.59, *p* = 0.010) and medical conditions (chi-square = − 2.654, *p* = 0.008) as the non-continuous variables were also significantly different between the PTSD SDPs and non-PTSD SDPs, indicating that the PTSD SDPs might have poorer health.

### The characteristics of the dead children and their impacts on the rate of PTSD in SDPs

Table 2 lists the rate of PTSD according to the characteristics of the dead children such as the gender, age, and the cause of death. No significant difference in PCL-C was found in the two Shidu groups divided by the gender of dead children, but groups divided by the causes of the children deaths-diseases and accidence, had significant difference in the PCL-C scores (Z = − 2.195, *p* = 0.028). SDPs of the lost adolescent child whose age was under 18, had significant different rate of PTSD compared to those with the adult child dead (Z = − 2.111, *p* = 0.035).

**Table 2:** The dead children characteristics on the rate of PTSD in SDPs

	N	PCL-C	Z	p
Gender			−0.926	0.923
Male	87	42.67 ± 17.41		
Female	62	42.82 ± 16.11		
Cause			−2.195	0.028*
Disease	107	40.94 ± 17.12		
Accident	42	47.29 ± 15.19		
Age			−2.111	0.035*
Adolescent	39	37.87 ± 16.55		
Adult	110	44.45 ± 16.60		

*Note.* PCL-C=Post-trauma stress disorder checklist-civilian version; * means *p* < 0.05

### The correlation between the factors of potential effects and PTSD

Table 3 illustrates the correlations between the above-mentioned factors including the demographic information and the characteristics of the dead child. The significant relationships with PTSD were observed in gender, income, age of being Shidu, annual hospital visit, medical conditions, years of being Shidu, age and causes of dead child (*p* value of all the factors less than 0.05). In general, being female, old, and unhealthy Shidu cohort presented a higher score in the PCL-C, which was supported by the existing positive Spearman correlation coefficient between PTSD and gender, age of being Shidu, hospital visit and medical conditions. Conversely, income and the years of being Shidu demonstrated the negative correlation to PTSD meaning that in our cohort SDPs with high income and long time of bereavement had low scores on PCL-C scale. As for the characteristics of the dead child, child age at death and the cause for death positively significantly correlated to parents’ PTSD severity, explaining the accident death of elder single child may generate severe PTSD in parents. It is notable that the positive correlation among age of being Shidu, years of being Shidu and age of dead child indicated that with the increasing age of the dead child, the age of parents for being Shidu were older and the time for recovering from loss was shorter. Additionally, as expected, the medical conditions were positively correlated to the annual hospital visit times (with the corerlation at 0.440).

**Table 3:** The Spearman correlation coefficient between the potential factors and PTSD

	1	2	3	4	5	6	7	8	9	10	11	12	13
1.PTSD	1												
2.Gender	.330**	1											
3.Income	−.219**	−.239**	1										
4.Age	−.079	−.316**	.318**	1									
5.Age of being Shidu	.166*	−.13	.165*	.520**	1								
6.Marriage	.058	.071	−.071	−.045	−.061	1							
7.Education	.046	−.063	.089	−.062	−.138	.052	1						
8.Annual hospital visit	.331**	.317**	−.045	−.043	.026	.025	−.15	1					
9.Medical conditions	.276**	.138	.042	.097	.133	−.012	−.056	.440**	1				
10.Years of being Shidu	−.221**	−.046	.001	.078	−.769**	.009	.067	−.028	−.086	1			
11.Gender of dead child	−.008	.04	−.098	−.187*	−.145	.011	−.103	.058	−.073	.065	1		
12.Age of dead child	.218**	−.025	.07	.315**	.874**	.017	−.111	.123	.076	−.784**	−.144	1	
13.causes	.180*	.019	−.055	.109	.007	.047	.085	.042	−.062	.111	.045	−.004	1

Note: *PTSD* Post-trauma stress disorder

* means *p* < 0.05 and ** means *p* < 0.01

### Risk factors of parents and the dead child predicting PCL-C scores

With the pattern of significant results of the univariate analyses as guidance, two hierarchical logistical regression analyses were conducted to examine the ability of risk factors. In the first step, parent gender, income, annual hospital visit, medical conditions and years of being Shidu were entered. On the second step, the child age at death and cause of death were entered. In the regression analysis, the dependent variable was the PCL-C scores, which were dichotomized by the rule that below 50 points was listed as 0 and not less than 50 points as 1. As presented in Table 4, gender, annual hospital visit and years of being Shidu were significant contributors, predicting 20.4% of the total variance in PCL-C scores (Chi-square = 22.29,*p* = 0.004). The entry of variables on a second step significantly increased the PCL-C variance explained, however, only the causes of the dead child was significantly predictive (*p* = 0.027) to 3.7% of the variance in PCL-C scores. However, in the step 2, years of being Shidu failed to account for the significant amount of unique variance. Besides, among all the significant variables, gender seemed to a key factor in the predicting analysis (OR = 2.877, 95% CI = 1.153~7.178), while the predictive effects of the characteristics of the dead children were less than the self condition of SDPs.

**Table 4:** Logistic regression analysis of risk factors of parents and the dead child predicting the PTSD in Shidu older adults

Model		B	Std. Error	Wald	Sig.	Exp(B)	95%CI		Adjusted R^2^
							Lower	upper	
1	(Constant)	−1.417	.893	2.516	.113	.242			.204**
	Gender (male = 0,femal = 1)	1.039	.46	5.096	.024*	2.827	1.147	6.971	
	Income (lower = 0,higher = 1)	−.774	.407	3.624	.057	.461	.208	1.023	
	Annual hospital visit	.762	.37	4.228	.04*	2.142	1.036	4.426	
	Medical conditions	.006	.206	.001	.977	1.006	.672	1.507	
	Years of being Shidu	−.363	.182	3.988	.046*	.696	.487	.993	
2	(Constant)	−1.654	1.074	2.372	.124	.191			.241***
	Gender (male = 0,femal = 1)	1.057	.466	5.131	.024*	2.877	1.153	7.178	
	Income (lower = 0,higher = 1)	−.769	.408	3.547	.06	0.464	.208	1.032	
	Annual hospital visit	.808	.376	4.612	.032*	2.243	1.073	4.689	
	Medical conditions	−.025	.21	.014	.907	.976	.646	1.474	
	Years of being Shidu	−.375	.206	3.29	.07	0.688	.459	1.031	
	Age of dead child	.095	.52	.034	.753	1.1	.397	3.046	
	Causes (disease = 0,accident = 1)	.615	.431	2.039	.027*	1.851	.795	4.307	

a. Dependent Variable: Non-PTSD = 0, PTSD = 1 b. * means *p* < 0.05, ** means *p* < 0.01, *** means *p* < 0.001

## Discussion

In our cohort of SDPs, the morbidity of PTSD reached 30.9% according to the clinical cut-off of 50 in PCL-C scale, while in the group of age over 60 the morbidity reached 31.6% (the average score of PCL-C was 42.65) and 28.6% in the group aged below 60. There was no significant difference between the two group as SDPs all aged over 49 close to the acknowledged average age for the old people in China. In fact, Chinese women usually retire at 50 and men at 55, therefore, the group aged from 49 to 59 are deemed as the subgroup of old people who are facing the challenge of aging or undergoing aging. As the result showed, the morbidity of PTSD was about twice higher in Shidu older adults compared to that reported in the bereaved old people who lost the spouse in an old age [17]. It emphasizes that loss of the only child is the bitterest distress to old people within the realm of bereavement [27]. Especially, in China, familism culture is a collectivist culture and has a profound effect on Chinese society, where the parent-child bonds are much strained [4]. A Chinese saying states that ‘losing one’s parent(s) in one’s youth, losing one’s spouse in middle-age, and losing one’s child in old age’ are the three greatest tragedies in life. Therefore, combined with the statistics, Shidu older adults might experience more unforgettable grieving process and higher vulnerability to PTSD, and, undoubtedly, deserve more assistance and help from the psychologists and social workers.

To explore the potential risk factors of PTSD in SDPs, two main influential factors were analyzed: the different characteristics of the parents and the deceased child based on the previous similar research of PTSD related to bereavement of child. The results showed the gender of SDPs was a stronger predictor of PTSD, indicating females had larger possibility of PTSD after the loss of their only child, which was consistent with the previous findings of parents’ bereavement [28, 29]. In a traditional Chinese family, mothers are expected to feed and take good care of their only child, and spend more time and love in the child than fathers, so mothers form a closer bond with their child, reflected in another Chinese proverb- “A begging mother is better than an official father”. Therefore, losing the only child produced more burden and stress for mothers than for fathers [11]. The other stronger predictor was the annual hospital visits which was significant in the two-step regression model. However, the medical conditions which were represented by the total number of the physical diseases were not significant in the regression. It could be explained by that a mental health state might be greatly related to the Shidu older adults’ vulnerability to PTSD since a lot of previous studies reported other mental disorders like depression [20], anxiety and insomnia usually coexisted in the bereaved people with PTSD [30, 31], which were significantly associated with the hospital visit time. However, in our study, the risk factors regarding PTSD in Shidu older adults by differentiating the symptoms were our focuses, instead of emerging the symptoms profiles from PTSD, prolonged grief disorder or depression, with the aim at yielding important information about PTSD profiles of distress following loss. Different from the other studies, the associations with PTSD among SDPs’ characteristics of education, marriage, and religion were not significant, which might partly owe to the local sampling. Although the income level was negatively related to PCL-C scores, it had tiny contribution to the possibility of having PTSD. As for the characteristics of the dead child, only the cause of death turned to be significantly contributed to the prevalence of PTSD. Notably, SDPs of child died from accidents have more possibility of PTSD than those of child died from diseases. It was documented a sudden and violent loss of a loved one can adversely affect mental health and grief in a substantial number of the bereaved [9]. PTSD were more severe after sudden and violent losses than losses following natural deaths, and the trajectory of recovery seemed to be slower [31]. A large national study showed the mothers whose children died from unnatural causes had an overall hazard ratio of 1.72, whereas the corresponding figure for those whose children died from natural causes was 1.33, reporting a higher hazard ratio in mothers whose children died unexpectedly [32]. Murphy et al. studied 175 bereaved parents for 5 years following three types of violent deaths: accidents, homicides, and suicides [9]. The report supported that the sudden, violent deaths of children produce particularly negative outcomes for the parents. As presumed by Floyd et al., the deaths that are violent or unexpected (e.g., accidents, homicide) could cause severe turmoil and emotional distress that overtax coping resources and produce more negative bereavement outcomes than deaths from non-violent, expected circumstances (e.g., long-term illness) [33]. However, a lot of studies of children died from cancer showed the possibility of having PTSD in bereaved parents are inconclusive, as the follow-up results diverse, but few of the studies are reported the elder SDPs’ chronic PTSD prevalence. Considering this, we investigated the years of being Shidu as a potential related factor of PTSD. and attained negative association between the time and PTSD in first-step regression. However, after the characteristics of the dead children entered, the contribution of years of being Shidu was not significant. Referred to the previous studies [10], the insignificant result might be due to the unknown relationship between the age and the cause of the child death, since the children with fatal diseases usually died at a young age and the parents have a long time to suffer from the distress, gradually developing into chronic PTSD, while a child died suddenly or unexpectedly usually in a older age resulting in the early PTSD. Further studies and expanded samples should be helpful to test the role in terms of the years of being Shidu. Besides, in line with other studies [34], there was no proof for significant effect of sex of the dead child on morbidity in these Shidu older adults, however, the results might be different if this study is replicated in a population with a different grief culture and, more importantly, different gender schemas.

The current study has some limitations. First, the sample size might not be enough, due to the difficult access to the group, to show some other factors’ significant relation to the prevalence of PTSD, and restricted the explanation. It should be aware that SDPs went through a lot of bitterness, for example, the broken marriage, low economical statue, discrimination from the society and so on. Interaction effect of these ordeals with the loss grief may be difficult to control. Considering this, we used a question in our demographic investigation asking-“Have you been through traumatic events in the last year”, to avoid the influence of the complicated grief. However, it may not be effective as the long-term grief could not be easily excluded. Second, the morbidity of PTSD in Shidu older adults in the present study might not exactly reflect the actual situation as some participant withdrew from the interview, who could have more severe PTSD. Third, the R square of the regression was small, which indicates more potential factors were undiscovered or the interactions of these factors were not significantly verified. Cooperated with result from a latent profiles of physical and psychological outcomes of bereaved parents in China who lost their only child [35], indicators could include subjective physical health, negative psychological outcomes and positive psychological outcomes. However, it should be highlighted that in our study the instrument of PTSD was PCL-C in Chines version which adopted a three-factor model of measuring the disorder, different from the Posttraumatic Stress Disorder Checklist for DSM-5 (PCL-5) formalized in 2013, which provided a four-factor model and high psychometric quality. Therefore, the contribution of the tested factors would be underestimated if more symptoms should be considered as indicating the probability of PTSD. Therefore, the future researches are still needed to study the PTSD of Shidu older adults with the new diagnostic instrument. Due to the limited effort, a more comprehensive assessment including the prolonged grief disorder and the potential additional losses has not been implemented in the study. However, a key distinctive feature of PGD is “yearning for the deceased”, whereas “fear” is the hallmark symptom of PTSD [36]. Recent researches have indicated that PGD may strongly overlap with Persistent Complex Bereavement Disorder, which is included in the Diagnostic and Statistical Manual of Mental Disorders, 5th edition as a condition requiring further research [37]. PGD is proposed as a separate construct in the DSM-5, and PGD symptoms strongly affect PTSD symptoms at the first year of a loss of loved one [38]. However, PGD has still not been studied in the context of SDPs and not an extrinsic factor in predicting PTSD in SDPs. Essentially, the comorbidity of depression and PGD could make the diagnosis of PTSD more intricate. Lastly, a longitudinal investigation of the sample may help to understand the high initial PTSD frequency, and help to identify who suffers from the long-term consequences of being old-age SDPs. Undoubtedly, this will be a hard work. In these elaborate works of our study, it has been found home visits to older participants is a more appropriate, although costly, way of obtaining responses of a satisfying data quality. Therefore, future study may benefit from paying for home visits to all participants both in the PTSD and the non-PTSD group,which is also advocated by O’Connor [27]. Despite these limitations, the results are noteworthy because this is the first study to evaluate PTSD in the Shidu older adults and explored the risk factors. In the future, more works should be done for the Shidu older adults such as the comorbidity of PTSD in them, other intermediates and moderators.

Despite these limitations, the present study makes several significant contributions to the knowledge on the sequelae of losing the only child. First, no previous cross-section study has reported on the prevalence of PTSD in Shidu older adults, while this study has found it was significantly higher than the other bereavement-related PTSD. These findings emphasize that Shidu is a major public health issue as a large group of Shidu parents in the coming century and the number is still increasing and suggest that screening assessments of PTSD in older SDPs might be useful in the identification of high-risk individuals for early interventions. Second, the current study has found a number of significant predictors of PTSD in SDPs. The results are somewhat consistent with the evidence about the predictors of PTSD after other types of bereavement. Especially, our results has implied women with frequent hospital visits, no matter for physic or mental health checks, who lost her child in an unexpected accidence could be a suffer of PTSD and need timely and substantial psychological help. Importantly, the overlap of predictors of psychiatric problems, like complicated grief and depression founded in studies about bereavement, has indicated the imperative intervention on these factors should be implemented. In turn, it would also be beneficial to combine the therapies which are targeting for the complicated grief and depression, in the treatment of PTSD in SDPs. Finally, the causes of child’s death were found to have the significant association with PTSD in SDPs. The accidence resulting in the unexpected death of the only child was a strong traumatic exposure to parents. Therefore, after-accidence psychological first aid should be promptly demanded and important. Overall, the results provide strong suggestive evidence that useful interventions could be developed in future prospective studies to target the prevention and treatment of PTSD in SDPs.

## Conclusion

With the accelerated process of aging, especially in China with rapid economical development, the Shidu older adults have become a group with more attentions. PTSD is clearly a possible reaction to the loss of the only child for considerable older bereaved people. In our study, the gender and hospital visits of the Shidu older adults and the causes of their child’s death have significant correlation to the prevalence of PTSD, which could help to improve the possibility of early identification of the high-risk Shidu older adults with PTSD, and providing timely and effective interventions. In the future, more elaboration is required to expand the study scale and to explore the undiscovered potential risk factors for the completion of the predicting profile of PTSD in the Shidu older adults.

## Data Availability

The datasets analyzed during the current study are not publicly available but are available from the corresponding author on reasonable request (may require data use agreements to be developed).

## Abbreviations

DSM-IV-TR: Diagnostic and Statistical Manual of Mental Disorders, Fourth Edition, Text Revision
PCL-C: PTSD Checklist–Civilian Version
PGD: Prolonged Grief Disorder
PTSD: Post Traumatic Stress Disorder
SDPs: Shidu parents
